# Factors associated with diabetes-related distress over time among patients with T2DM in a tertiary hospital in Singapore

**DOI:** 10.1186/s12902-017-0184-4

**Published:** 2017-06-23

**Authors:** Maudrene L. Tan, Chuen S. Tan, Konstadina Griva, Yung S. Lee, Jeannette Lee, E. S. Tai, Eric Y. Khoo, Hwee-Lin Wee

**Affiliations:** 1 Saw Swee Hock School of Public Health, National University of Singapore, Singapore, Singapore; 2 Department of Psychology, National University of Singapore, Singapore, Singapore; 3 Department of Pediatrics, Yong Loo Lin School of Medicine, National University of Singapore, Singapore, Singapore; 4 Division of Paediatric Endocrinology and Diabetes, Khoo-Teck Puat-National University Children’s Medical Institute, National University Health System, Singapore, Singapore; 5 Singapore Institute for Clinical Sciences, Agency for Science, Technology and Research, Singapore, Singapore; 6 Department of Medicine, National University Health System, Singapore, Singapore; 7 Department of Pharmacy, National University of Singapore, 10 Kent Ridge Crescent, Singapore, 119260 Singapore

**Keywords:** Diabetes-related distress, Diabetes, Longitudinal, Excessive daytime sleepiness

## Abstract

**Background:**

Persistent diabetes-related distress (DRD) is experienced by patients with Type 2 Diabetes Mellitus. Knowing factors associated with persistent DRD will aid clinicians in prioritising interventions efforts.

**Methods:**

A total of 216 patients were recruited from a tertiary hospital in Singapore, an Asian city state, and followed for 1.5 years (2011–2014). Data was collected by self-completed questionnaires assessing DRD (measured by the Problem Areas in Diabetes score) and other psychosocial aspects such as social support, presenteeism, depression, health-related quality of life (HRQoL) and excessive daytime sleepiness (EDS) at three time points. Clinical data (body-mass-index and glycated haemoglobin) was obtained from medical records. Change score was calculated for each clinical and psychosocial variable to capture changes in these variables from baseline. Generalized Linear Model with Generalized Estimating Equation method was used to assess whether baseline and change scores in clinical and psychosocial are associated with DRD over time.

**Results:**

Complete data was available for 73 patients, with mean age 44 (SD 12.5) years and 67% males. Persistent DRD was experienced by 21% of the patients. In the final model, baseline HRQoL (OR = 0.56, *p* < 0.05) and change score of EDS (OR = 1.22, *p* < 0.05) was significantly associated with DRD over time.

**Conclusions:**

EDS might be a surrogate marker for persistent DRD and should be explored in larger samples of population to confirm the findings from this study.

## Background

Persistent diabetes-related distress (DRD), which is defined as distress experienced over at least nine months [1] has been shown to lead to clinical depression [2]. Lipscombe et al. found that baseline factors such as being older, not married, having more complications and chronic conditions, having less family support and being depressed were associated with increasing and severe levels of distress [3].

The aim of this study is to explore changes in clinical (such as body mass index (BMI) and glycated haemoglobin (HbA1c)) and psychosocial factors (such as social support, presenteeism, depression, health-related quality of life (HRQoL) and excessive daytime sleepiness (EDS)), above and beyond baseline, and their association with DRD over time. Observed significant changes will enable researchers and clinicians to prioritise their interventions [4] to prevent the development of persistent DRD.

## Methods

This is a longitudinal study on psychological outcomes in patients with diabetes mellitus with data collected over three time-points (baseline “B”, first follow-up “F1” at 6 months and second follow-up “F2” between 12 and 18 months) conducted at a single tertiary hospital in Singapore [5]. Only patients with type 2 diabetes mellitus (T2DM) were included in this analysis. The institutional review board of the National Healthcare Group reviewed and approved the study protocol. Informed consent was obtained from all participating patients.

Socio-demographic information were collected using self-administered questionnaires, clinical factors, such as BMI, HbA1c and medication usage were retrieved from electronic medical records (EMR) and medical history of co-morbidities (i.e., cardiovascular disease, retinopathy, nephropathy, peripheral vascular disease, cerebrovascular disease and anaemia) were captured through a combination of self-report and EMR.

Distress was measured with the Problem Areas in Diabetes (PAID), with scores 40 and above indicative of severe DRD [6]. Social support was measured with two items: 1) single item measuring support from family using the Family Functioning Measure [7]; 2) Question 22 from the World Health Organisation Quality of Life Brief questionnaire measuring support from friends [8]. Presenteeism was measured using the modified question “On a scale of 0 “Least effective” to 10 “Most effective”, how effective are you at work?” [9]. Depression was captured using Kessler-10 Psychological Distress Scale [10]. HRQoL was measured with the Audit of Diabetes-Dependent Quality of Life [11]. EDS was measured using the Epworth Sleepiness Scale [12].

### Statistical analysis

Generalized linear model (GLM) with the Generalized Estimating Equation (GEE) method was used to model the longitudinal binary outcome. Three steps were taken to generate the final model: Step 1, we performed a regression analysis for each selected clinical and psychosocial variables at baseline with adjustment for time (i.e., baseline, follow-up 1 and 2) (M1). Step 2, on top of the baseline clinical or psychosocial variable from M1, we added its corresponding change score which captures its change from baseline (M2). In step 3, in addition to the variables in M2, we adjusted for baseline socio-demographic (Age, Gender: Male/Female, Ethnicity: Chinese/Non-Chinese), medication usage (Insulin: Yes/No) and comorbidities (Yes/No) (M3). Our final model included clinical and psychosocial variables which were significant in M3 and baseline socio-demographic, medication usage and comorbidities. As the change scores were considered higher order terms to the baseline variables, the baseline would be included into the final model although only the change score was significant.

All statistical analyses were performed using Stata 12.0 for Windows (Stata Corporation, College Station, Texas, USA).

## Results

Of the 216 subjects recruited at baseline, only 73 patients had complete data at three time-points from 2011 to 2014, resulting in an overall attrition rate of 66.2%. A comparison of subjects at baseline and those who did not return can be found in Table 1. The patients who did not return were not different from those at baseline in their clinical and psychosocial characteristics.

**Table 1:** Comparison of patients’ characteristics of those who did not return after the baseline visit and those who completed the entire study

Variables	Returned for all time points (*n* = 73)	Did not return for all time points (*n* = 143)	*p*-value
	%, Mean (sd)	%, Mean (sd)	
Social demographics factors			
Gender			0.23
Male	67.1	59.4	
Female	32.9	40.6	
Age (Years)	44.1 (12.5)	46.0 (12.0)	0.26
Ethnicity			0.95
Chinese	52.1	49.0	
Non-Chinese	48.0	51.1	
Clinical factors			
Presence of Comorbidities			0.52
No	32.9	28.7	
Yes	67.1	71.3	
Insulin use			0.13
No	69.9	56.0	
Yes	30.1	44.0	
Body Mass Index (kg/m2)	29.3 (5.1)	28.9 (5.8)	0.56
HbA1C (%)	8.2 (1.5)	8.3 (2.1)	0.59
Psycosocial factors			
Support from friends			0.48
No	33.3	28.7	
Yes	66.7	71.3	
Family Functioning Measure (FFM)			0.40
No	7.0	10.6	
Yes	93.0	89.4	
Presenteeism			0.46
No	10.0	13.6	
Yes	90.0	86.4	
ADDQoL (HRQoL)	6.4 (1.9)	5.9 (2.3)	0.13
Sleepiness	7.1 (5.2)	6.7 (5.3)	0.57
Depression	19.4 (6.3)	19.5 (7.3)	0.93
Psychological			
Problem Areas in Diabetes (PAID)			0.32
No, not distressed	67.1	60.1	
Yes, distressed	32.9	39.9	

Table 2 presented the results of the various models with varying degree of adjustment and the results were in general consistent in the direction of association among the significant findings. In the first model with adjustment for time only (M1), baseline HbA1c, support from friends, depression and HRQoL were significantly associated with DRD over time. When change scores were included into the models (M2), only baseline depression and baseline HRQoL remained significantly associated with current distress. Although baseline EDS remained insignificant in both M1 and M2, change in current EDS (from baseline) was significantly associated with DRD over time. The significant findings in M2 persisted after adjusting for baseline socio-demographic, medication use and comorbidities (M3). Interestingly, baseline HbA1c became significantly associated with DRD over time in M3 even though it was not significant in M2 but significant in M1.

**Table 2:** Clinical and psychological factors associated with current diabetes-related distress over time

	Model 1 (M1)			Model 2 (M2)			Model 3 (M3)		
	OR	CI	*p*-value	OR	CI	*p*-value	OR	CI	*p*-value
Clinical variables									
BMI kg/m^2^									
Baseline	1.09	(0.99, 1.20)	0.08	1.08	(0.92, 1.20)	0.20	1.12	(0.99, 1.26)	0.06
Change				0.98	(0.91, 1.05)	0.51	0.98	(0.91, 1.05)	0.51
HbA1c (%)									
Baseline	1.43	(1.02, 2.02)	**0.04**	1.38	(0.93, 2.06)	0.11	1.63	(1.02, 2.60)	**0.04**
Change				1.15	(0.92, 1.45)	0.22	1.18	(0.93, 1.51)	0.17
Psychosocial variables									
Support from family									
Baseline	0.56	(0.28, 1.12)	0.10	0.58	(0.29, 1.17)	0.13	0.62	(0.31, 1.24)	0.18
Change				1.11	(0.70, 1.77)	0.66	1.24	(0.73, 2.13)	0.43
Support from friends									
Baseline	0.44	(0.19, 0.98)	**0.05**	0.45	(0.20, 1.04)	0.06	0.49	(0.17, 1.37)	0.17
Change				1.12	(0.83, 1.50)	0.47	1.18	(0.88, 1.58)	0.28
Presenteeism									
Baseline	1.03	(0.67, 1.58)	0.90	1.03	(0.67, 1.59)	0.90	1.07	(0.67, 1.69)	0.79
Change				1.00	(0.78, 1.28)	1.00	0.99	(0.76, 1.29)	0.92
Depression									
Baseline	1.15	(1.05, 1.26)	**<0.01**	1.16	(1.06, 1.27)	**<0.01**	1.21	(1.05, 1.41)	**<0.01**
Change				1.06	(0.98, 1.14)	0.14	1.04	(0.94, 1.15)	0.43
HRQoL									
Baseline	0.57	(0.43, 0.75)	**<0.01**	0.55	(0.42, 0.74)	**<0.01**	0.53	(0.38, 0.74)	**<0.01**
Change				0.89	(0.61, 1.30)	0.56	0.90	(0.58, 1.41)	0.65
EDS									
Baseline	1.07	(0.97, 1.18)	0.20	1.09	(0.98, 1.20)	0.10	1.05	(0.94, 1.18)	0.36
Change				1.13	(1.05, 1.22)	**<0.01**	1.16	(1.05, 1.27)	**<0.01**

Bold figures indicate statistical significance with *p* < 0.05

The final model (Table 3) included all significant clinical and psychological variables from M3. Baseline HRQoL and change score of EDS remained significantly associated with DRD over time, where higher HRQoL at baseline significantly reduced the odds of DRD (OR = 0.60, *p* < 0.01) over time but increase in EDS from baseline over time was significantly associated with increased odds in DRD over time (OR = 1.22, *p* < 0.01).

**Table 3:** Final model for diabetes-related distress over three time-points

	OR	95% CI	*p*-value
Visit			
Follow-up 1	0.68	(0.26, 1.80)	0.44
Follow-up 2	1.20	(0.72, 2.03)	0.48
Female	0.67	(0.18, 2.56)	0.56
Age	1.04	(0.97, 1.12)	0.25
Non-Chinese	0.93	(0.28, 3.07)	0.91
Insulin: Yes	0.33	(0.08, 1.31)	0.12
Comorbidities: Yes	1.01	(0.28, 3.72)	0.98
Baseline HbA1c	1.27	(0.80, 2.02)	0.31
Baseline HRQoL	0.56	(0.42, 0.76)	**<0.01**
Baseline Depression	1.12	(0.97, 1.29)	0.13
Baseline EDS	1.05	(0.94, 1.17)	0.37
Change in EDS from baseline	1.22	(1.07, 1.38)	**<0.01**

*Abbreviations*: *HbA1c* Glycated Haemoglobin, *HRQoL* Health-Related Quality of Life, *EDS* Excessive Daytime Sleepiness

Bold figures indicate statistical significance with *p* < 0.05

## Discussion

In this study, we found that increase in current EDS from baseline, was significantly associated with increased odds of DRD over time, suggesting the compound negative impact of EDS on DRD with time. Possible explanations for this include: 1) EDS has been shown to be associated with reduced motivation to engage in diabetes self-management activities [13], which ultimately results in poorer control [14] and hence DRD [15]; 2) EDS being positively associated with depression [16], which has also shown to be associated with DRD [17]; and 3) EDS is associated with sleep disorders such as obstructive sleep apnea (OSA), restless leg syndrome (RLS) and insomnia [18] which have been shown to negatively affect HbA1c [14], further aggravating the DRD. This suggests that EDS can be used as a surrogate marker for DRD and can be addressed in interventions that aims to reduce DRD.

Of particular interest was the finding that persistent DRD was only associated with psychosocial rather than clinical variables in our study. These findings corroborated with previous studies which showed that psychosocial variables had a larger impact on DRD over time compared to clinical factors [19, 20]. One postulation is that clinical variables are indicators of the emotional stresses experienced by patients [20], since patients with diabetes may suffer from distress due to the disease and the challenges from the daily grind of living, managing and caring for diabetes [21]. Another possibility is related to the concept whereby patients’ behaviours are primarily driven by their own perceptions and their surrounding influences [22]. Given that T2DM is a chronic condition which involves a life-long process of complex and demanding set of self-management instructions, DRD is likely to be driven by patients’ perceptions rather than the disease status of the patients. This would suggest the importance of behavioural intervention to help patients cope with DRD.

Based on a literature review on PUBMED, no studies were found to have addressed the association between EDS and DRD. However, studies have shown the association between EDS and depression [16, 23]. In fact, Koutsourelakis et al noted that depression and diabetes were key determinants of EDS among those with OSA [24]. As such, it is possible that the relationship between EDS and DRD is bidirectional. To factor in the possibility of reversal causality in our findings, we reanalysed Model 3 with DRD as a continuous variable and modelled both baseline and change in EDS as endogenous variables that were functions of DRD. The results from the reanalysis were similar to Table 3, suggesting the robustness of the significant association between changes in EDS and DRD, and clinicians should consider addressing EDS in routine clinic sessions.

While this study presents novel findings, a major limitation faced was the large attrition of patients from the cohort. This not only severely limited the power of the analysis, but could have introduced bias in the findings. However, when we compared the clinical and psychosocial characteristics between those who returned for the follow-ups and those who did not, there were no significant differences between the two groups (Table 1) suggesting bias in the findings might be minimal.

Second, we did not collect additional information on sleep phenotype, apart from the Epworth Sleepiness Scale. In patients with T2DM, EDS has been shown to be associated with sleep disorders such as OSA, RLS and insomnia [18, 25, 26]. However, the study by Dixon et al has shown that EDS was more associated with poor energy, depression and symptoms of nocturnal sleep disturbance than OSA in obese patients [27]. Nonetheless, future work addressing the issue of sleep in patients with T2DM should include other measures of sleep to have a better understanding on how sleep affects the emotional aspects of patients with T2DM.

Last, our sample was drawn from a specialist outpatient clinic of the National University Hospital thus limiting the generalizability of our findings.

## Conclusion

Increased EDS, above and beyond baseline, and baseline HRQoL were associated with persistent DRD. Given that the change in EDS appears to have an impact on persistent DRD in our study, clinicians could consider looking out for signs of EDS during routine clinical practice to address persistent DRD. However, in light of the limited sample size of this study, it is important for larger cohorts to study the effect of EDS on DRD to confirm the findings.

## Abbreviations

BMI: Body mass index
DRD: Diabetes-related distress
EDS: Excessive daytime sleepiness
EMR: Electronic medical records
GEE: Generalized estimating equation
GLM: Generalized linear model
HbA1c: Glycated haemoglobin
HRQoL: Health-related quality of life
OSA: Obstructive sleep apnea
PAID: Problem areas in diabetes
PUBMED: Search engine accessing primarily the MEDLINE database of references and abstracts on life sciences and biomedical topics
RLS: Restless leg syndrome
T2DM: Type 2 diabetes mellitus
